# Supplementary material for: Interleukin-35 Prevents Development of Autoimmune Diabetes Possibly by Maintaining the Phenotype of Regulatory B Cells
Source: Int J Mol Sci. 2021 Nov 30;22(23):12988. doi: 10.3390/ijms222312988 (PMC8657454; doi:10.3390/ijms222312988)
Supplement: Supplementary file 1 [file ijms-22-12988-s001.zip › ijms-1471551-supplementary.pdf]

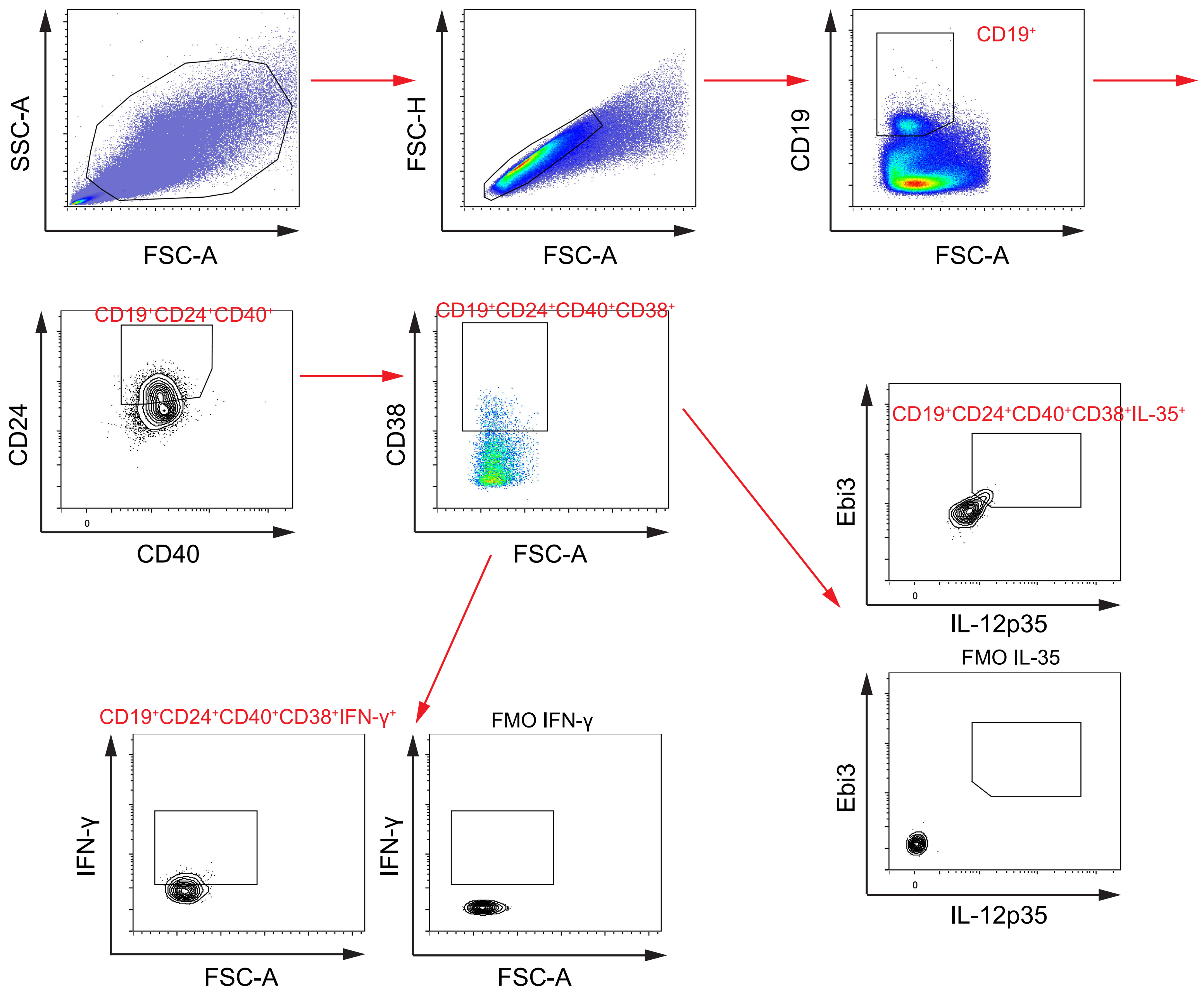

Supplementary Figure S1

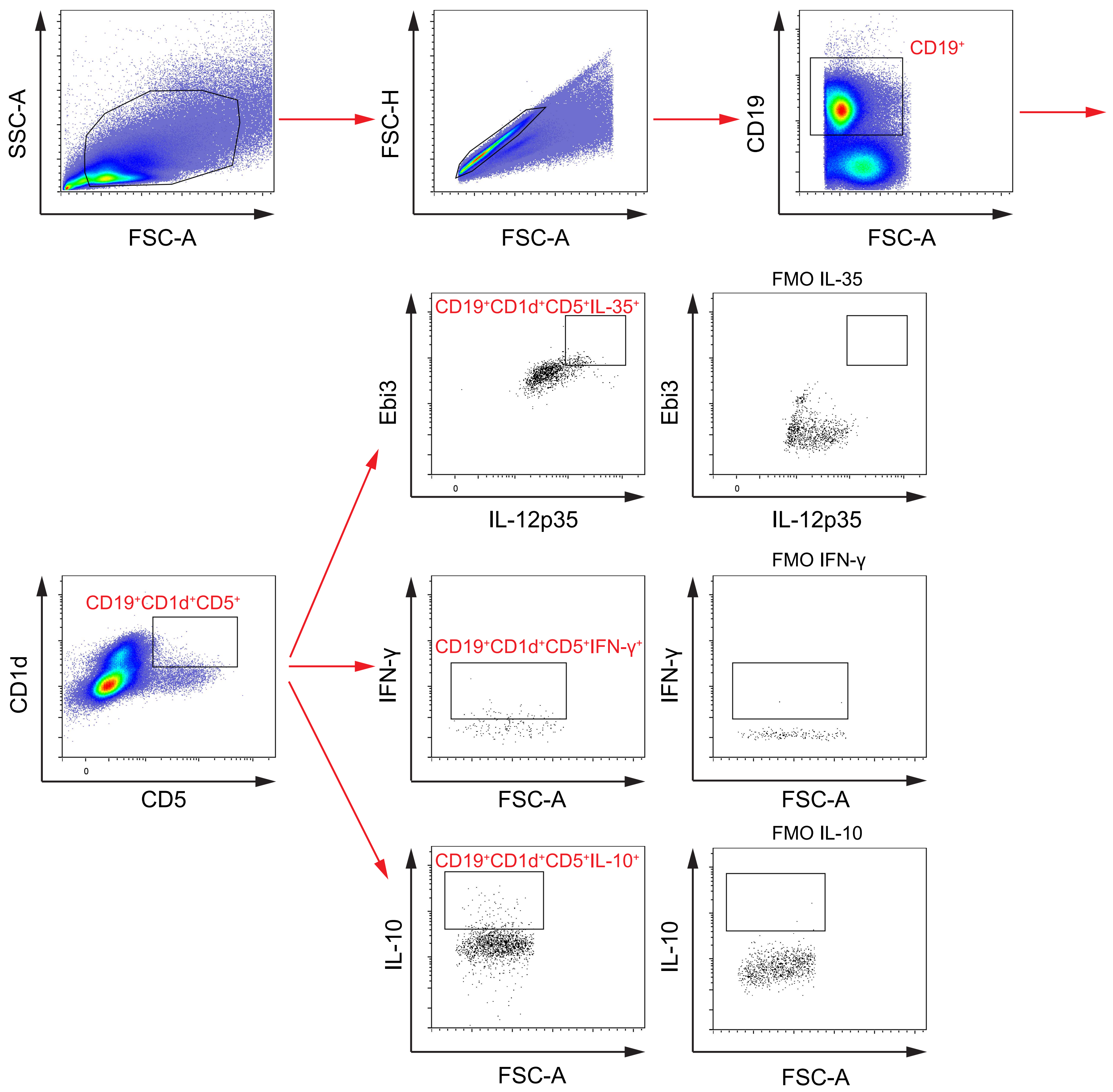

Supplementary Figure S2

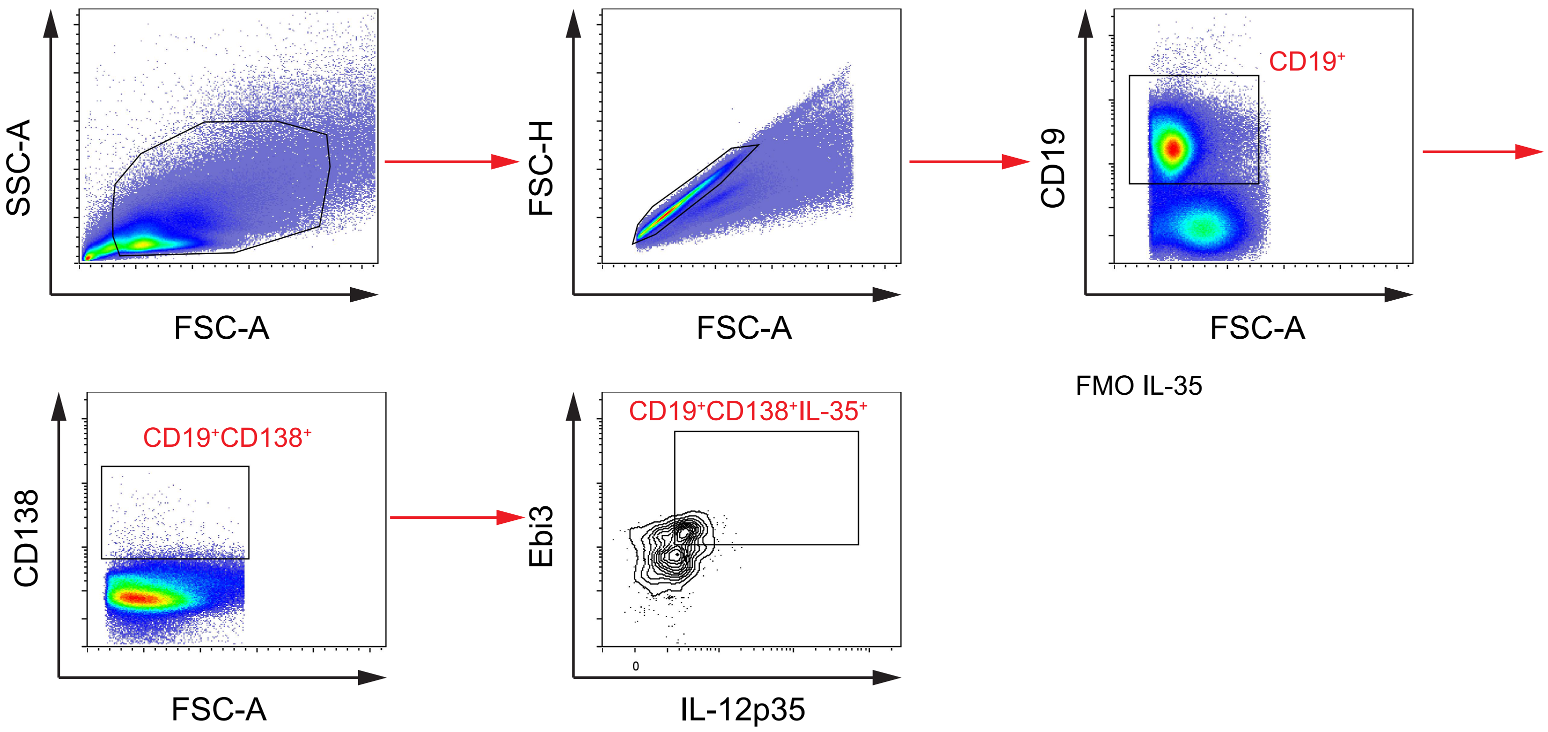

Supplementary Figure S3

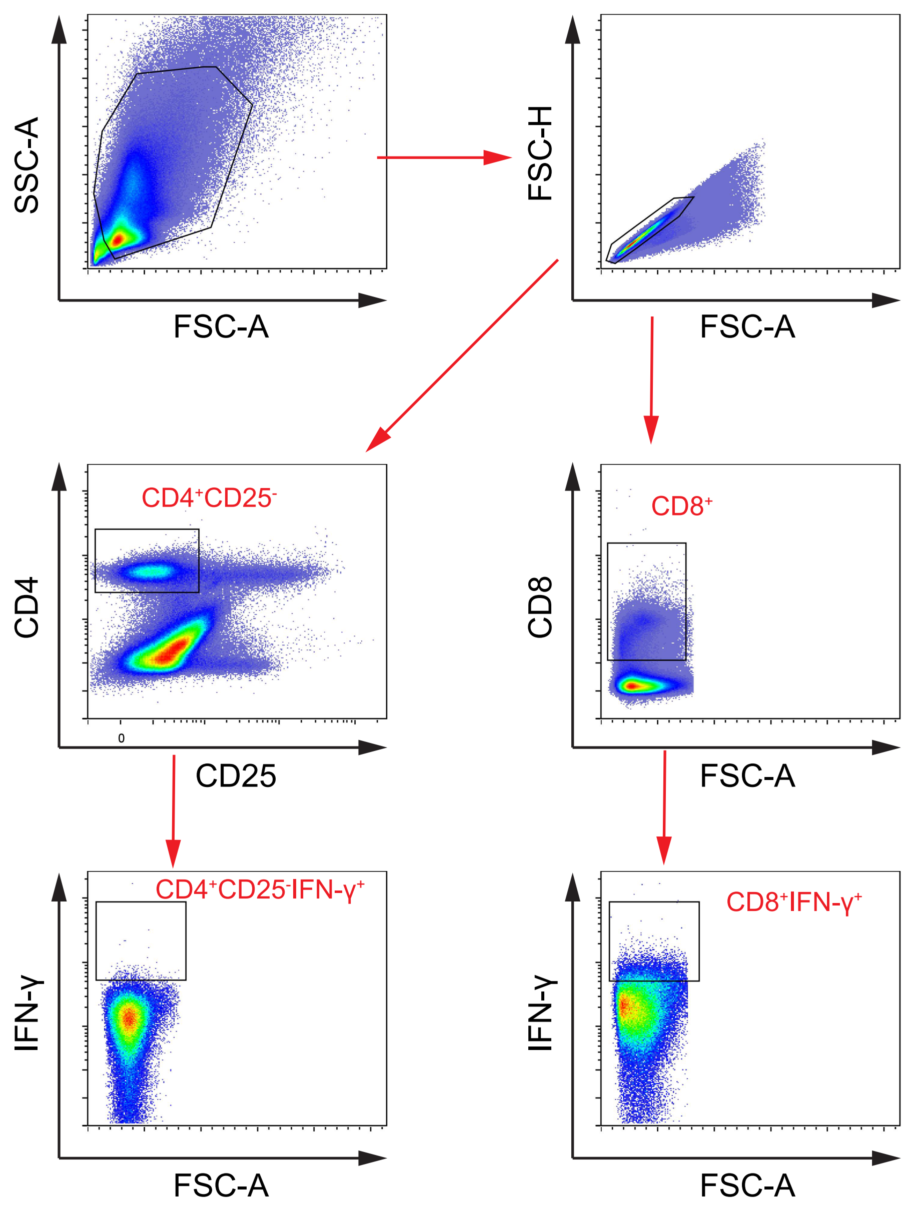

Supplementary Figure S4

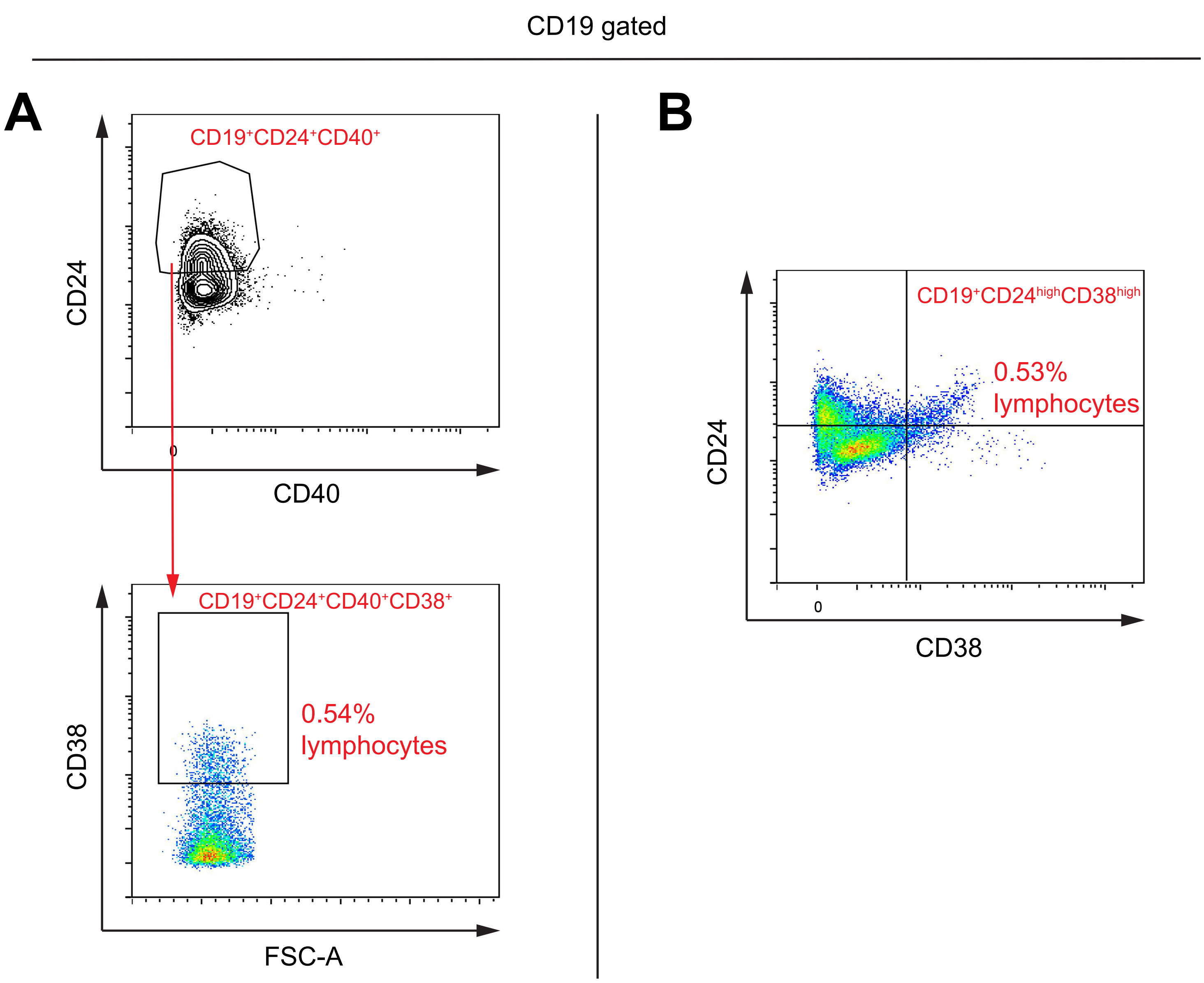

Supplementary Figure S5

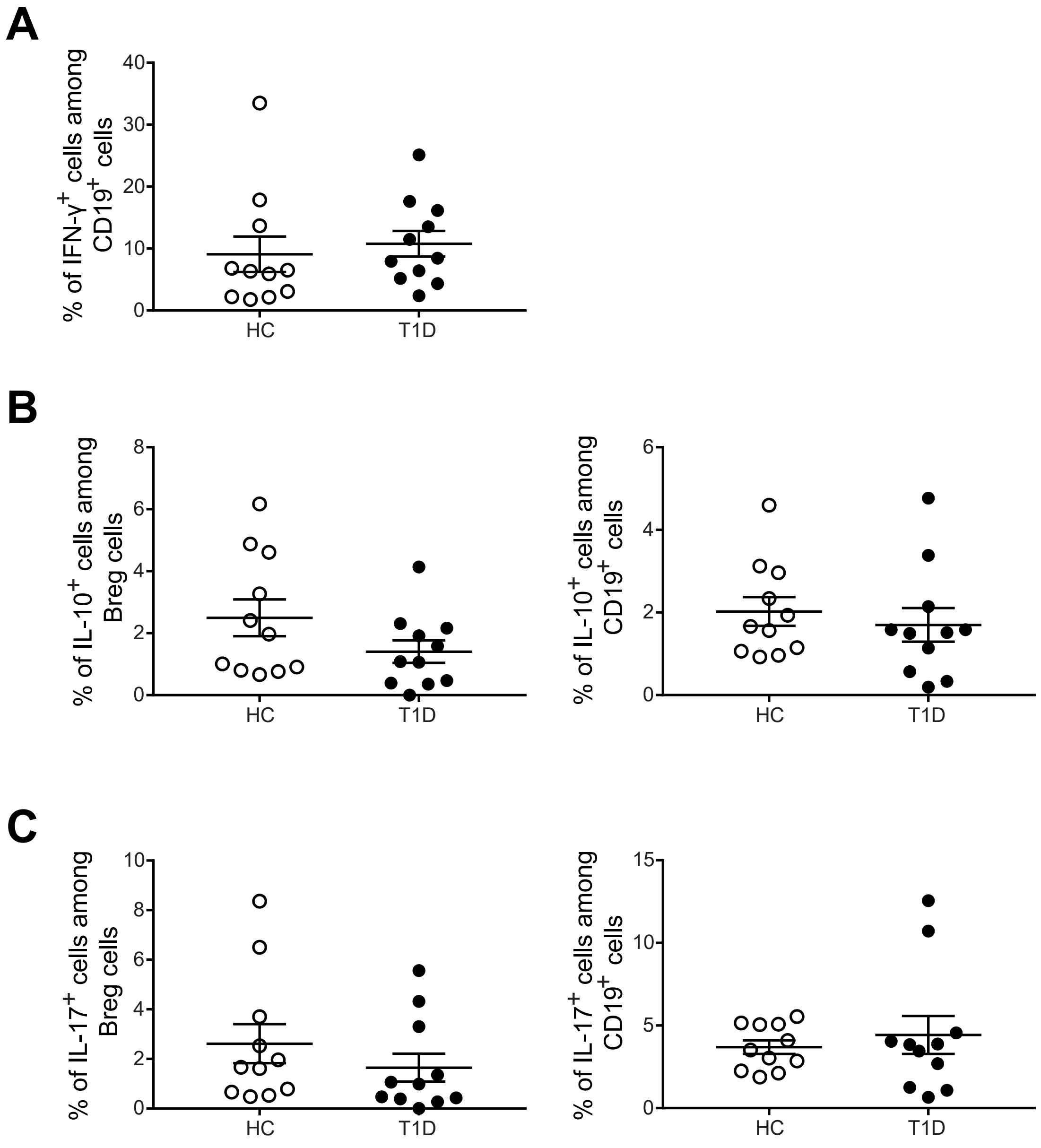

Supplementary Figure S6

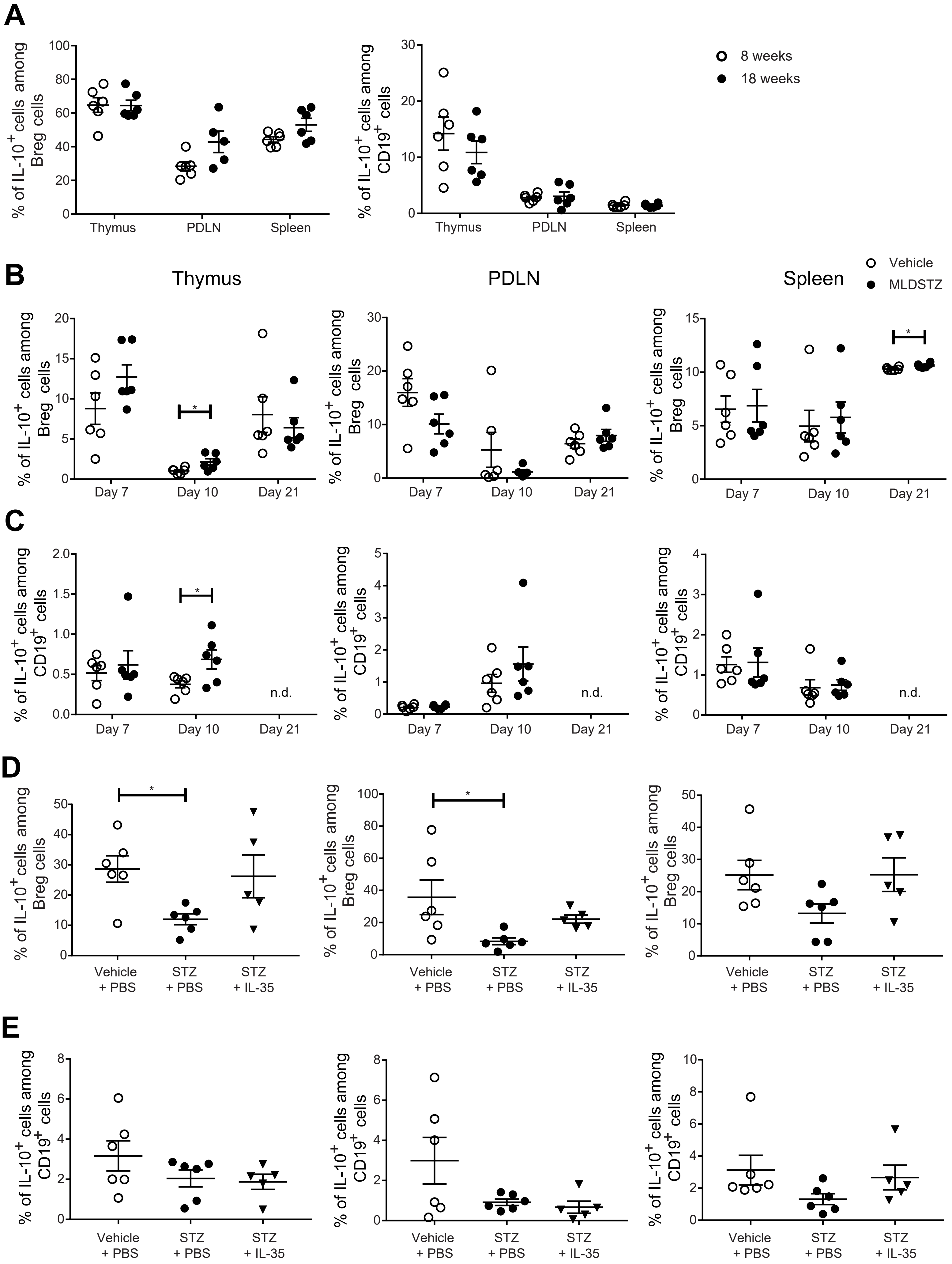

Supplementary Figure S7

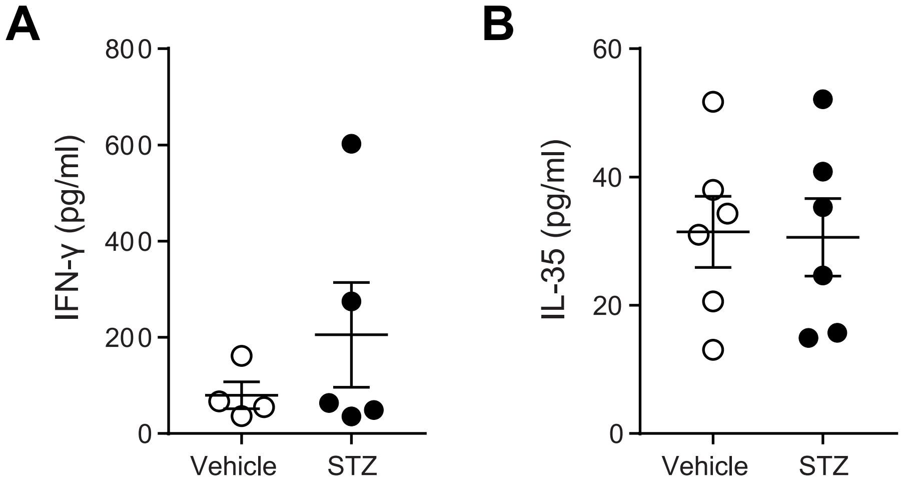

Supplementary Figure S8

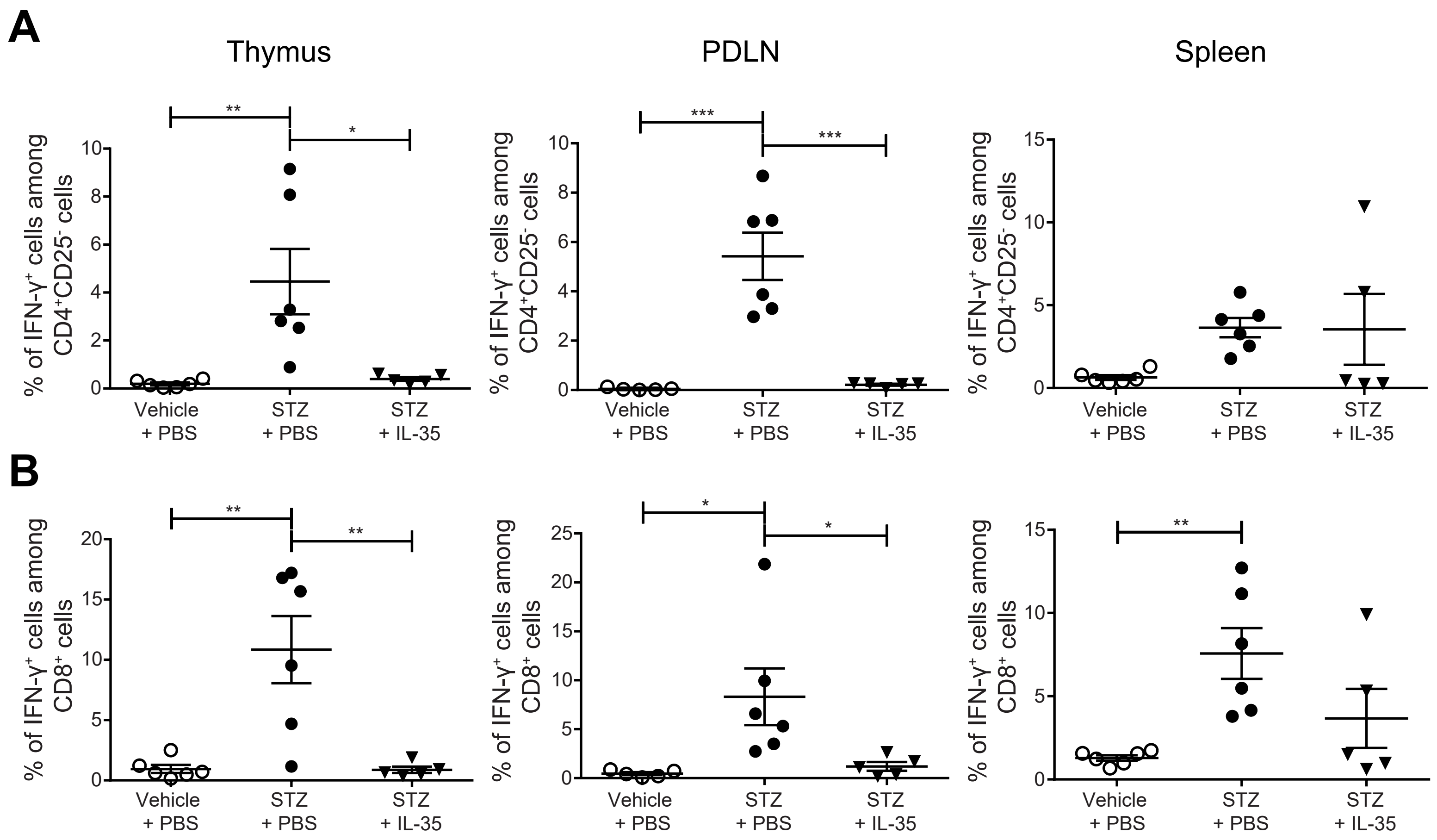

Supplementary Figure S9

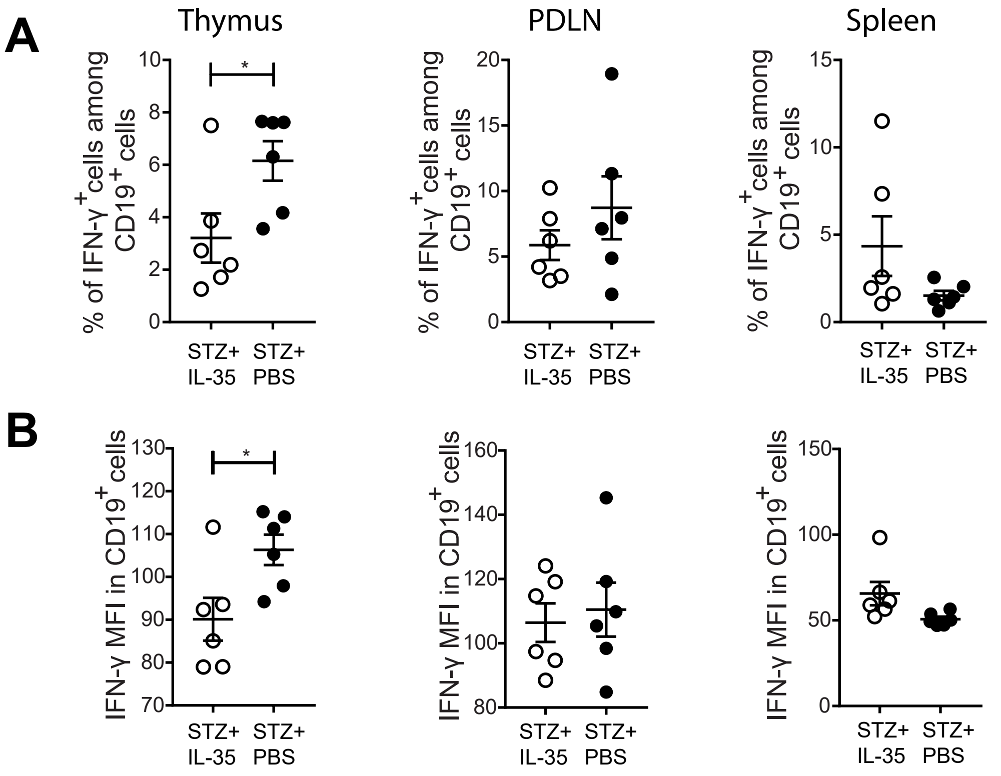

Supplementary Figure S10

Supplementary Figure S1. Representative gating strategies for human Breg cells. The live cells were gated based on forward scatter (FSC) and side scatter (SSC). Cells were further gated for CD19 expression. $CD19^+$ cells were thereafter gated for the expression of CD24 and CD40. $CD19^+CD24^+CD40^+CD38^+$ cells were gated for Ebi3 and IL-12p35 expression or IFN-γ expression. Gates were drawn by using fluorescence minus one controls to analyze $IL\text{-}35^+$ and $IFN\text{-}\gamma^+$ cells.

Supplementary Figure S2. Representative gating strategies for mouse Breg cells. The live cells were gated based on forward scatter (FSC) and side scatter (SSC). Cells were further gated for CD19 expression. $CD19^+$ cells were thereafter gated for the expression of CD1d and CD5. $CD19^+CD1d^+CD5^+$ cells were gated for Ebi3 and IL-12p35 expression, IFN-γ or IL-10 expression. Gates were drawn by using FMO controls to analyze $IL\text{-}35^+$, $IFN\text{-}\gamma^+$ and $IL\text{-}10^+$ cells.

Supplementary Figure S3. Representative gating strategies for mouse plasma cells. The live cells were gated based on forward scatter (FSC) and side scatter (SSC). Cells were further gated for CD19 expression. $CD19^+$ cells were then gated for the expression of CD138. $CD19^+CD138^+$ cells were gated for Ebi3 and IL-12p35 expression.

Supplementary Figure S4. Representative gating strategies for mouse T cells. The live cells were gated based on forward scatter (FSC) and side scatter (SSC). Cells were further gated for CD4 and CD25 expression or CD8 expression. $CD4^+CD25^-$ cells and $CD8^+$ cells were then gated for the expression of IFN-γ.

Supplementary Figure S5. Comparison of gating strategies for human Breg cells. $CD19^+$ cells were gated for CD24 and CD38 expression using our gating strategy (A) or gating strategy reported in ref. 11 and ref.22 (B). Analyses using both gating strategies yielded similar results.

Supplementary Figure S6. Proportions of IFN- γ^+, $IL\text{-}10^+$ and $IL\text{-}17^+$ cells in the PBMCs of T1D subjects and HC. PBMCs were isolated from peripheral blood of subjects with T1D and HC. Cell

proportions were determined by flow cytometry. (A) Proportions of IFN- γ^+ cells among $CD19^+$ cells. (B) Proportions of IL-10^+ cells among Breg and $CD19^+$ cells. (C) Proportions of IL-17^+ cells among Breg and $CD19^+$ cells. Results are expressed as means ± SEM. Unpaired t tests were performed for comparison.

Supplementary Figure S7. Proportions of IL-10^+ Breg cells in NOD and MLDSTZ mice. 8-week-old and 18-week-old female NOD mice were killed, single cells were isolated from removed thymi, PDLNs and spleens. Cells were then stained and analyzed using flow cytometry. (A) Proportions of IL-10^+ cells among Breg cells and IL-10^+ Breg cells in NOD female mice. Male CD-1 mice were injected with saline or low dose STZ for 5 consecutive days. Mice were killed on days 0, 7, 10 and 21 after the first STZ injection, organs were treated with the same manners as for NOD mice. (B) Proportions of IL-10^+ cells among Breg cells. (C) Proportions of IL-10^+ cells among $CD19^+$ cells. Male CD-1 mice were injected with saline or low dose STZ for 5 consecutive days, and for the next 8 consecutive days with PBS or recombinant mouse IL-35. (D) Proportions of IL-10^+ cells among Breg cells. (E) Proportions of IL-10^+ cells among $CD19^+$ cells. Results are expressed as means ± SEM, from two experiments (n = 2-3 mice/group/experiment). Unpaired t tests were performed for comparison.

Supplementary Figure S8. IFN-γ and IL-35 concentration in B cell culture supernatants. Male CD-1 mice were injected with saline or low dose STZ for 5 consecutive days. $CD19^+$ B cells from mice killed on day 21 were cultured for 72 h with 0.1 mg/ml CD40L and 1 μl/ml LPS. 50 ng/ml PMA and 1 μM ionomycin were added 5 h before harvesting the cells. Culture supernatants were saved for ELISA. (A) Concentrations of IFN-γ. (B) Concentrations of IL-35. Results are expressed as means ± SEM, from two experiments (n = 2-3 mice/group/experiment). Unpaired t tests were performed for comparison.

Supplementary Figure S9. Proportions of IFN-γ$^+$ T cells among CD4$^+$CD25$^-$ and CD8$^+$ cells are decreased in STZ mice treated with IL-35. Male CD-1 mice were injected with saline or low dose STZ for 5 consecutive days, and for the next 8 consecutive days with PBS or recombinant mouse IL-35. Single cells were isolated from removed thymi, PDLNs and spleens. Cells were then stained and analyzed using flow cytometry. (A) Proportions of IFN-γ$^+$ cells among CD4$^+$CD25$^-$ cells. (B) Proportions of IFN-γ$^+$ cells among CD8$^+$ cells. Results are expressed as means ± SEM, from two experiments (n = 2-3 mice/group/experiment). One-way ANOVA followed by Tukey's test were performed for comparison. *, ** and *** denote $p < 0.05$, $p < 0.01$ and $p < 0.001$, respectively.

Supplementary Figure S10. Proportions of IFN-γ$^+$ T cells among CD4$^+$CD25$^-$ and CD8$^+$ cells are decreased in STZ mice treated with IL-35. After receiving STZ treatment for 5 consecutive days, male CD-1 mice received treatment with PBS or recombinant mouse IL-35 for 8 consecutive days. Single cells were isolated from removed thymi, PDLNs and spleens. Cells were then stained and analyzed using flow cytometry. (A) Proportions of IFN-γ$^+$ cells among CD19$^+$ cells. (B) Mean fluorescence intensities of IFN-γ in CD19$^+$ cells. Results are expressed as means ± SEM, from two experiments (n = 2-3 mice/group/experiment). Unpaired t tests were performed for comparison. * denotes $p < 0.05$.
